# A novel grade-lymph node ratio model predicts the prognosis of the advanced gastric cancer patients after neoadjuvant radiotherapy

**DOI:** 10.18632/oncotarget.12573

**Published:** 2016-10-11

**Authors:** Jianjun Liu, Mingxue Su, Jing Wang, Gan Zhang, Jing Zhou, Anqing Zhang, Zixue Ren, Xucai Zheng, Shikai Hong, Shengying Wang, Rongxin Zhang

**Affiliations:** ^1^ Department of Head - Neck and Thoracic Surgery, Anhui Provincial Cancer Hospital, West branch of Anhui Province Hospital, Hefei, China; ^2^ Department of Infectious Disease Epidemiology, Luan Peoples Hospital, Luan, China; ^3^ Department of Urologic Surgery, Anhui Provincial Cancer Hospital, West branch of Anhui Province Hospital, Hefei, China

**Keywords:** neoadjuvant radiotherapy, gastric cancer, survival analysis, SEER

## Abstract

Although local advanced gastric cancer (AGC) could benefit from neoadjuvant radiotherapy (NRT), there are few studies evaluating patients survival after NRT. In current study, we aimed to investigate the value of prognostic factors in AGC patients after NRT and to evaluate whether post-therapy pathological characteristics were predictive factors in these patients. We retrospectively analyzed AGC patients who underwent NRT from Surveillance, Epidemiology, and End Results (SEER) Database. The patients clinical and post-therapy pathological characteristics were analyzed. The best cutoff points for continuous variables were identified by X-tile. The discrimination of risk factors were compared by receiver operating characteristic (ROC) curve. As a result, 1,429 AGC patients were included into this study. In the multivariate analysis, the lymph nodes status and histology grade were significant risk factors for DSS (disease special survival). Then, we propose a novel Grade-lymph node Ratio (G-R) staging system for the AGC patients survival prognosis. Clearly, the new G-R staging system has a more-accurate 3-year and 5-year DSS prediction than the AJCC staging system (*p* = 0.001, 0.007, respectively). In conclusions, the current large, general population-based study demonstrated that the G-R staging system resulting in more-accurate DSS prediction. It could be regarded as a reliable classification for AGC patients after NRT in future.

## INTRODUCTION

Gastric cancer is the fourth most common malignancy and second leading cause of cancer-related death worldwide. [1] In 2014, approximately 22,220 new cases were diagnosed and 10,990 deaths attributed to gastric cancer in United States. Although the incidence has declined recently, the 5-year survival was less than 30%. [2, 3]

Completed resection with lymph node dissection is the only potential curative treatment for resectable cancer. [4] However, the cancer symptoms usually were not obvious in early stage, most patients were diagnosed with advanced stage in most countries. Recently, the MAGIC trials demonstrated that the neoadjuvant chemotherapy improved survival for AGC patients. [5] Meanwhile, several phase I/II trails also demonstrated a survival benefit for AGC patients after NRT. [6–8] Indeed, the neoadjuvant therapy can achieve a clinical downstaging before resection and increase the possibility of R0 resection for AGC patients. [9–11] As a result, the neoadjuvant therapy were widely used for potentially resectable AGC before surgery.

Based on those promising results, the neoadjuvant therapy may be a potential standard treatments for AGC patients. However, the prognostic value of post-therapy pathologic characteristic were still unclear, especially in patients underwent NRT. [10, 12–15] In current study, we aimed to investigate the value of prognostic factors in AGC patients and to evaluate whether post-therapy pathological characteristics were predictive factors of survival in these patients.

## RESULTS

### Patients and demographics

A total of 1,429 patients between January 1998 and November 2013 in the SEER database who met all the inclusion criteria were analyzed in current study. The patients’ characteristics were listed in Table 1. Overall, there were 1,206 male patients and 223 female patients, and the median age was 60.8 years old. There were also 60 (4.2%) patients who received adjuvant radiotherapy after curative resection. The mean number of positive lymph node was 1.8±3.3, and the mean total examined number of lymph node was 14.3±9.9. With a median follow-up of 31.9 months, there were 760 (53.2%) patients died before the analysis of the present study, and 102 (13.4%) patients of them were died because of other causes.

**Table 1:** Characteristic of patients from SEER database

Characteristic	Patients(*n*= 1,429)	
	NO.	%
Age (years)		
Median	60.8±10.4	
Range	14 to 88	
Gender		
Male	1206	84.4
Female	223	15.6
Race		
White	1278	89.4
Black	61	4.3
AI	18	1.3
API	70	4.9
Unknown	2	0.1
Tumor size (cm)		
Median(*n* = 1027)	4.6±3.4	
Range	0.1 to 50	
Tumor location		
Cardia	1299	90.9
Fundus	9	0.6
Body	17	1.2
Antrum	32	2.2
Pylorus	4	0.3
Lesser curvature	29	2.0
Greater curvature	9	0.6
Overlapping	18	1.3
Unknown	12	0.8
Grade		
Well differentiated	62	4.3
Moderately differentiated	510	35.7
Poorly differentiated	827	57.9
Undifferentiated	30	2.1
Depth of invasion		
Mucosa or submucosa	134	9.4
Proper muscle	215	15.0
Subserosa	667	46.7
Serosa	260	18.2
Adjacent invasion	125	8.7
Unknown	28	2.0
Number of positive LN.		
0	757	53.0
1 to 2	342	23.9
3 to 6	231	16.2
7 to 15	81	5.7
16 or more	18	1.3
Positive LN (Mean±SD)	1.8±3.3	
Total LN (Mean±SD)(n=1411)	14.3±9.9	
AJCC Stage		
IA	100	7.0
IB	146	10.2
IIA	410	28.7
IIB	307	21.5
IIIA	196	13.7
IIIB	173	12.1
IIIC	69	4.8
Unknown	28	2.0
Adjuvant radiotherapy		
Yes	60	4.2
No	1369	95.8

Abbreviations: AI, American Indian or Alaska Native; API, Asian or Pacific Islander; LN, lymph node; AJCC, American Joint Committee on Cancer.

**Table 2:** Prognostic factors for DSS of all the patients

	Univariate analysis			Multivariate Analysis		
	HR	95% CI	*p*	HR	95% CI	*p*
Age	1.005	0.998 to 1.013	0.167			
Gender (Female / Male)	0.879	0.708 to 1.091	0.243			
Race		0.062				
API/ nonAPI	0.762	0.573 to 1.014				
Location			0.939			
Antrum	ref					
Fundus	0.771	0.220 to 2.707				
Body	1.255	0.520 to 3.029				
Cardia	1.336	0.771 to 2.315				
Pylorus	1.464	0.417 to 5.139				
Lesser curvature	1.462	0.678 to 3.155				
Greater curvature	1.371	0.488 to 3.847				
Overlapping	1.547	0.661 to 3.620				
Grade			<0.001			0.010
Well /Moderately differentiated	0.714	0.608 to 0.838		0.807	0.686 to 0.950	
Poorly differentiated/ Undifferentiated						
Total LN	1.002	0.994 to 1.010	0.584			
Tumor Size (*n* = 1027)	1.001	0.999 to 1.004	0.414			
Depth of invasion			0.017			0.638
Mucosa or submucosa	ref					
Proper muscle	1.106	0.797 to 1.535				
Subserosa	1.068	0.800 to 1.424				
Serosa	1.304	0.964 to 1.765				
Adjacent invasion	1.549	1.105 to 2.173				
mLNR stage.			<0.001			<0.001
0	ref			ref		
1	1.522	1.233 to 1.879		1.496	1.209 to 1.851	
2	2.378	1.910 to 2.962		2.295	1.834 to 2.872	
3	3.413	2.779 to 4.191		2.269	2.648 to 4.036	
Adjuvant radiotherapy (ART/ no-ART)	0.948	0.795 to 1.129	0.546			

Abbreviations: HR:hazard ratio;API, Asian or Pacific Islander; LN, lymph node; mLNR: metastastic lymph node ratio; ART: Adjuvant radiotherapy.

### Survival and lymph node ratio categories

**Figure 1:**
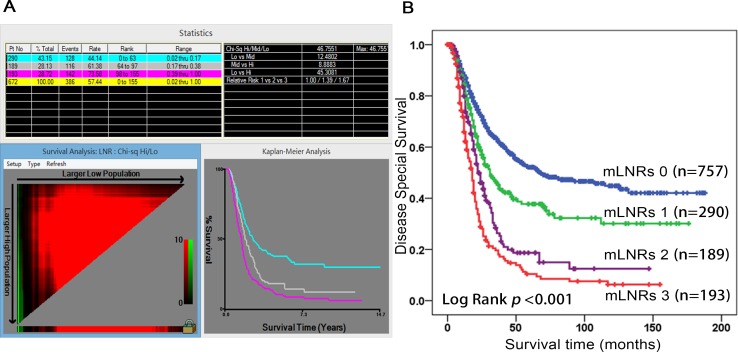
X-tile analysis identified the best cutoff points for mLNR A., and validated by Kaplan-Meier Curve B Abbreviation: mLNR, metastatic lymph node ratio.

The median DSS for all patients was 35 months, and the 1-year DSS, 3-year DSS, 5-year DSS were 83.0%, 48.9% and 39.0%, respectively. Lymph node ratio was defined as the number of positive nodes divided by the total examined nodes. As shown in the Figure 1, the best cutoff points for mLNR were 17.0% and 38.0%. Therefore, the mLNR were classified into four groups, as the following intervals. The mLNRs 0: mLNR = 0%; mLNRs 1: 0% < mLNR < 17.0%; mLNRs 2: 17.0% < mLNR < 38.0%; mLNRs 3: 38.0% < mLNR < 100%. 5-year DSS for the 4-level mLNR were 52.1%, 36.8%, 14.8% and 10.4%, respectively.

### Analysis of post-therapy prognostic factors

In the univariate analysis, the depth of invasion, grade and mLNRs were significant risk factors for AGC after NRT, whereas, other factors such as age, gender, race, location, total examined lymph node (TLN) were not correlated with DSS. However, in the multivariate analysis, the depth of invasion lost statistical significance as a prognostic factor (*p* = 0.638).

**Figure 2:**
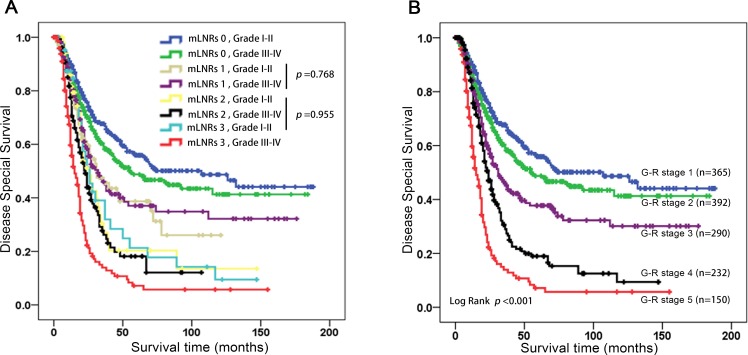
A. The patients were grouped into 8 groups and validated Kaplan-Meier Curve. B The patients were grouped into 5 groups and validated Kaplan-Meier Curve. Abbreviation: mLNRs, metastatic lymph node ratio stage; G-R stage, Grade-lymph node Ratio.

According to the Cox regression analysis, we divided the patients into 8 groups (Group 1: mLNRs 0 and Grade I-II; Group 2: mLNRs 0 and Grade III-IV; Group 3: mLNRs 1 and Grade I-II; Group 4: mLNRs 1 and Grade III-IV; Group 5: mLNRs 2 and Grade I-II; Group 6: mLNRs 2 and Grade III-IV; Group 7: mLNRs 3 and Grade I-II; Group 8: mLNRs 3 and Grade III-IV) based on grade and mLNRs (Figure 2A). However, as shown in Figure 2A, there was no significant difference between Group 3 and 4 (log rank *p* = 0.768), and also have insignificant difference in Group 5, 6 and 7 (log rank *p* = 0.955). As a result, we propose a novel Grade-lymph node Ratio (G-R) staging system for all the AGC patients after NRT (As shown in Figure 2B). The G-R 1 was defined as mLNR equal to 0% and Grade I-II; G-R 2 as mLNR equal to 0% and Grade III-IV; G-R 3 as mLNRs 1; G-R 4 as mLNRs 2, or mLNRs 3 with Grade I-II; G-R 5 as mLNRs 3 and Grade III-IV. The observed 5-year DSS curves based G-R staging was shown in Figure 2B.

### Comparison of predictive accuracy for G-R staging system single independent factors and AJCC staging system

**Figure 3:**
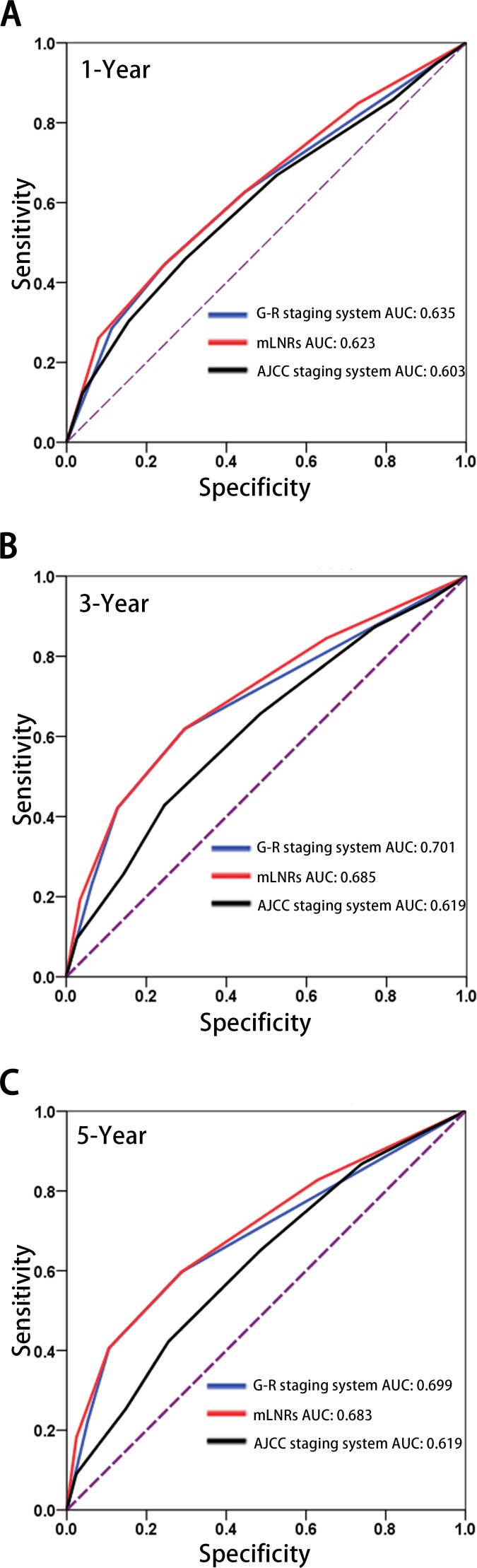
Comparison for the AUC of G-R staging system predicted, AJCC staging system and mLNRs to preidciton DSS at 1-yearA., 3-yearB., 5-yearC Abbreviation: AUC, areas under the receiver operating curves; mLNRs, metastatic lymph node ratio stage; G-R stage, Grade-lymph node Ratio; DSS, disease special survival.

The Area Under the ROC Curve (AUC) values were used to compare the discrimination for the G-R staging system and other prognostic models (mLNRs, AJCC staging system). The statistics power for discrimination between G-R staging system and AJCC staging system were compared in each time points. As shown in Table 3 and Figure 3, the AUC of G-R staging system were higher than mLNRs and AJCC staging system in both 3-year and 5-year time points (*p* = 0.001, 0.007, respectively). Whereas, the AUC of G-R staging system were higher than AJCC staging system 1-year DSS but did not reach statistical significance (*p* = 0.282).

**Table 3:** Comparison of predictive accuracy of DSS for G-R staging system, single independent factor and the 7th AJCC staging system in each time points

Time points	G-R staging system		mLNR stage		AJCC staging system		
	AUC	95%CI	AUC	95%CI	AUC	95%CI	*
1-Year	0.635	0.592 to 0.679	0.623	0.579 to 0.666	0.603	0.559 to 0.647	0.282
3-Year	0.701	0.668 to 0.735	0.685	0.651 to 0.720	0.619	0.583 to 0.656	0.001
5-Year	0.699	0.661 to 0.738	0.683	0.644 to 0.721	0.619	0.575 to 0.662	0.007

* :The *p* value in the AUC were the G-R staging model compared with AJCC staging system.

Abbreviations: DSS, disease special survival; AUC, Area Under the ROC Curve; AJCC, American Joint Committee on Cancer.

## DISCUSSION

In the present study, we developed a novel G-R staging system for AGC patients after NRT. A total of 1,429 AGC patients who received surgery following NRT from SEER database between 1998 and 2012 were analyzed. We demonstrated that the new G-R staging system has a more-accurate 3-year and 5-year DSS prediction than the AJCC staging system (*p* value, < 0.001, 0.008, respectively).

The survival benefit of neoadjuvant therapy had been investigated in various tumors, including esophageal, pancreatic and rectal cancers. [16–18] However, the prognostic value of post-therapy pathological characteristics in gastric cancer is still controversial. In 1999, Andrew ML et al analyzed 83 western AGC patients who were treated with neoadjuvant chemotherapy form three phase II trials. [13] With 26 months follow-up, the authors demonstrated that all the post-therapy T-N-M stage were not correlated with patients’ survival. In contrast, Kazumasa FJ et al analyzed 70 Asian AGC patients after neoadjuvant chemotherapy in 2012, and the authors identified that the post-therapy nodal status is significantly associated with patients’ overall survival. [12] In fact, indicators that independently and optimally reflect AGC patients’ survival who received NRT had not been discovered. In 2005, Ajani JA et al conducted a study to investigate prognosis factors for overall survival for AGC patients after neoadjuvant chemoradiotherapy. [14] A total of 41 AGC patients from M.D. Anderson Cancer Center were analyzed. The authors found that the post-therapy T-N-M stage was associated with overall survival. The findings were interesting, however, the samples were small, and the patients’ histological type had not been analyzed. In addition, all of the AGC patients in those studies were form tertiary care institutions that specialize in the treatment of malignancies, and the patients may have different characteristics from community institutions.

Currently, our study first review of a large data from national cancer registry, and established a novel G-R staging system for AGC patients after NRT. Although the conventional AJCC staging system represents an optimal tool in the field of gastric cancer, it is unclear whether this staging system suitable for gastric cancer patients after neoadjuvant therapy. [19] In fact, the known factor post-therapy depth of invasion after surgery held statistical significance in the univariate analysis, however, it did not maintain significance in the multivariate analysis (*p* = 0.638) which was consistent with previous studies. [12, 13] Therefore, the AJCC staging system based on depth of invasion, nodal status and metastatic status may not be applicable for AGC patients after NRT. In contrast, the histology grade and mLNR were the only two prognosis factors correlated with DSS both in univariate and multivariate analysis. Based on this findings, we established a novel classifier for AGC patients after NRT. The G-R staging system appeared to have a more-accurate DSS prediction than AJCC staging system or single independent factors. Since approximately 50% of the gastrectomy cases included in the SEER database came from community institutions, it is inconceivable that the G-R staging system has a broader applicability. [20]

The completed (R0) resection with D2 lymphadenectomy was the only potential curative for GC patients. [4] Since resection with D2 lymphadenectomy had not been widely performed in United States, the variable whether the patient received D2 lymphadenectomy was not included in this study. Additionally, although the previous studies demonstrated that the R0 resection was a significant risk for patients’ survival, such direct evidence is unavailable because that SEER database did not collect the information of gastrectomy surgical margin status. [21] Given that the current study was a retrospective study, only the patients who had complete information were included in present analysis, there may be a selection bias. In addition, in the univariate analysis and multivariate analysis, the number of positive lymph node was also associated with patient's survival. The discrimination of mLNRs was better than that of positive lymph node number, but the difference was insignificant (data not shown). Given that the positive lymph nodes number identified depends on the pathologic procedure and surgical scope, we used mLNRs instead of positive lymph nodes number in the new classifier. [22]

Since the G-R staging system have a more-accurate survival prediction, it would be favor for designing postoperative treatment, particularly, which stage patients can obtain survival benefit from adjuvant radiotherapy after surgery. However, the difference of adjuvant radiotherapy were not correlated with DSS in each G-R stages (detailed data unshown). However, these findings should be interpreted with caution. The reasons are as follows: Firstly, there is missing information regarding the use of adjuvant chemotherapy. It is possible that patients may have received adjuvant chemotherapy after surgery, resulting in a potential confounder in this study. Secondly, the possibility of selection bias could be acknowledged, since the current analysis was not based on a randomized patient population. Thirdly, since there were only 60 patients underwent adjuvant radiotherapy included in this study, it may lead to a bias result.

In conclusion, the current large, general population-based study demonstrated that the post-therapy pathological characteristics were associated with the survival of AGC patients after NRT. The post-therapy grade and mLNR were the only two predictors for those patients. Based on this finding, the novel G-R staging system showed optimal accuracy of survival prediction for AGC patients after neoadjuvant therapy. Through this classifier, clinicians can estimate the survival of the AGC patients after NRT more precisely.

## METHODS AND PATIENTS

### Surveillance, epidemiology, and end results (SEER) database

The SEER program, a large population-based collaboration program, was surveyed by the National Cancer Institute. A total of 18 population-based cancer registries were participated in this program, and the data are updated annually. It collects and provides approximately 26% American population's cancer incidence and survival data.

Gastric cancer (ICD-O-3 code within the range of 8000-8152, 8154-8231, 8243-8245, 8250-8576, 8940-8950, and 8980-8990) undergoing NRT between January 1998 and December 2012 were eligible for current study. The inclusion criteria were as follows: 1) Patients without distant metastasis; 2) patients received gastrectomy (Surgery code within the range of 30-80); 3) patients received radiotherapy before surgery.

### Statistical methods

The data of patients’ clinicpathological characteristics such as age at diagnosis, gender, race, marital status, surgery, tumor site, size, histology, grade, depth of invasion, number of positive lymph node (PLN) and TLN were collected. The primary endpoint was DSS, which was defined as the time form surgery to cancer-related death or the last follow-up. The pathological characteristics depth of invasion, lymph nodal stage and tumor stage were restaged according to the 7th edition AJCC staging system.

Tumor grade is a measurement of how closely the tumor cells resemble the normal gastric cancer. Well-differentiated (Grade I) and moderately-differentiated (Grade II) tumor cells closely resemble the tissue from normal gastric, whereas, poorly-differentiated (Grade III) and undifferentiated (Grade IV) tumor cells are disorganized and abnormal looking.

The mLNR was defined as the number of metastatic lymph nodes divided by the number of examined lymph nodes. A unique category mLNRs 0 was defined for patients with no regional positive lymph node, since those patients had significantly better prognosis than other patients (HR = 0.48, *p* < 0.001). The patients with mLNR higher than 0% were categorized by X-tile (http://www.tissuearray.org/rimmlab/). By this statistic tool, the continuous variables can divide into three groups. Associations between each group can be calculated by various standard statistical tests, including the log-rank test for survival and means tests for associations between other marker data. The X-tile can provide the optimal division of the data by p values obtained from a lookup table [23]

Independent risk factors were identified by the Cox regression analysis. DSS estimation and survival curves were performed by the Kaplan-Meier method and valided by the log-rank test. The Discrimination between the proposed G-R staging system and AJCC staging system was performed with ROC curve.

All analyses were performed by the software statistical package for social sciences (SPSS) version 19.0 (Chicago, IL), X-tile and the R software version 3.13 (http://www.r-project.org/). All the statistical tests were two sided. *p* value < 0.05 was considered to be statistically significant.
